# Contribution to the ecology of the Italian hare (*Lepus corsicanus*)

**DOI:** 10.1038/s41598-020-70013-1

**Published:** 2020-08-04

**Authors:** Maria Buglione, Simona Petrelli, Gabriele de Filippo, Claudia Troiano, Eleonora Rivieccio, Tommaso Notomista, Valeria Maselli, Luciano di Martino, Marco Carafa, Romano Gregorio, Roberta Latini, Mario Fortebraccio, Giorgia Romeo, Claudia Biliotti, Domenico Fulgione

**Affiliations:** 1 Department of Biology, University of Naples Federico II, Naples, Italy; 2 Istituto di Gestione della Fauna (IGF), Naples, Italy; 3 Department of Humanities, University of Naples Federico II, Napoli, Italy; 4 Majella National Park, Sulmona, Aquila Italy; 5 Cilento, Vallo di Diano e Alburni National Park, Salerno, Italy; 6 Abruzzo, Lazio and Molise National Park, Pescasseroli, Aquila Italy; 7 Freelance Forestry Doctor, Potenza, Italy; 8 Wildlife Section, Tuscan Regional Council, Grosseto, Italy; 9 SOS Animali Onlus, Wildlife Rescue Center, Semproniano, Grosseto Italy

**Keywords:** Biodiversity, Conservation biology, Zoology

## Abstract

The Italian hare (*Lepus corsicanus*) is endemic to Central-Southern Italy and Sicily, classified as vulnerable due to habitat alterations, low density and fragmented populations and ecological competition with the sympatric European hare (*Lepus europaeus*). Despite this *status*, only few and local studies have explored its ecological features. We provided some key traits of the ecological niche of the Italian hare as well as its potential distribution in the Italian peninsula. All data derived from genetically validated presences. We generated a habitat suitability model using maximum entropy distribution model for the Italian hare and its main competitor, the European hare. The dietary habits were obtained for the Italian hare with DNA metabarcoding and High-Throughput Sequencing on faecal pellets. The most relevant environmental variables affecting the potential distribution of the Italian hare are shared with the European hare, suggesting a potential competition. The variation in the observed altitudinal distribution is statistically significant between the two species.The diet of the Italian hare all year around includes 344 plant taxa accounted by 62 families. The *Fagaceae*, *Fabaceae*, *Poaceae*, *Rosaceae* and *Solanaceae* (counts > 20,000) represented the 90.22% of the total diet. *Fabaceae* (60.70%) and *Fagaceae* (67.47%) were the most abundant plant items occurring in the Spring/Summer and Autumn/Winter diets, respectively. The Spring/Summer diet showed richness (N = 266) and diversity index values (Shannon: 2.329, Evenness: 0.03858, Equitability: 0.4169) higher than the Autumn/Winter diet (N = 199, Shannon: 1.818, Evenness: 0.03096, Equitability: 0.3435). Our contribution adds important information to broaden the knowledge on the environmental (spatial and trophic) requirements of the Italian hare, representing effective support for fitting management actions in conservation planning.

## Introduction

The Italian hare (*Lepus corsicanus*) is an endemism of Central-Southern Italy and Sicily^1–5^, and shows a controversial but intriguing evolutionary history. The species was firstly stated as true by^6^, but later it was reconsidered to be a subspecies of the European hare (*L. europaeus*)^7^. After morphometric^8,9^ and molecular evidence^10^, it was re-evaluated as a true species, showing a long-lasting history of independent evolution from the European hare. Furthermore, some studies based on genetic^11–14^ and ecological^15^ data, suggested that *L. corsicanus* and *L. castroviejoi*, the broom hare, endemic of the Cantabrian Mountains of the Iberian Peninsula^16^, might be conspecific and sister taxa. In fact, despite the present allopatric distribution, both due to sharing of a common ancestor during the late Pleistocene^12^ or to hybridization and introgression events^11^, they could be considered two important evolutionary units^13^ to be preserved.

The Italian hare is classified as vulnerable (C2a1 category) according to the International Union for Conservation of Nature in its Red List^17^. The *status* of the species is affected mainly by habitat fragmentation, low population density, as well as the presence of small and isolated subpopulations^18,19^. Moreover, the Italian hare is also threatened by ecological competition with native and exotic lineages of the European hare, a cosmopolitan species intensively restocked as a game species^19–21^. Because of these reintroductions, there are some areas in Italy where the Italian hare and the European hare occur in sympatry^19,20,22–25^.

Currently, the most conspicuous populations of the Italian hare are living in association to protected areas, thanks to safeguarding policies active in these territories. Indeed, in Italy, the hunting plan provides only for the European hare to be hunted outside the territories included in the National Parks. However, the congeneric Italian hare and European hare are difficult to distinguish based only on their morphological characteristics^9^. This is particularly true in the wild, often leading to erroneous killing of the protected species with impoverishment of the populations outside the National Parks, where hunting is permitted.

Furthermore, the frequent sympatry between the Italian hare and the European hare^18,19,26^ could erroneously lead to the conclusion that they show a similar ecological demand.

**Figure 1:**
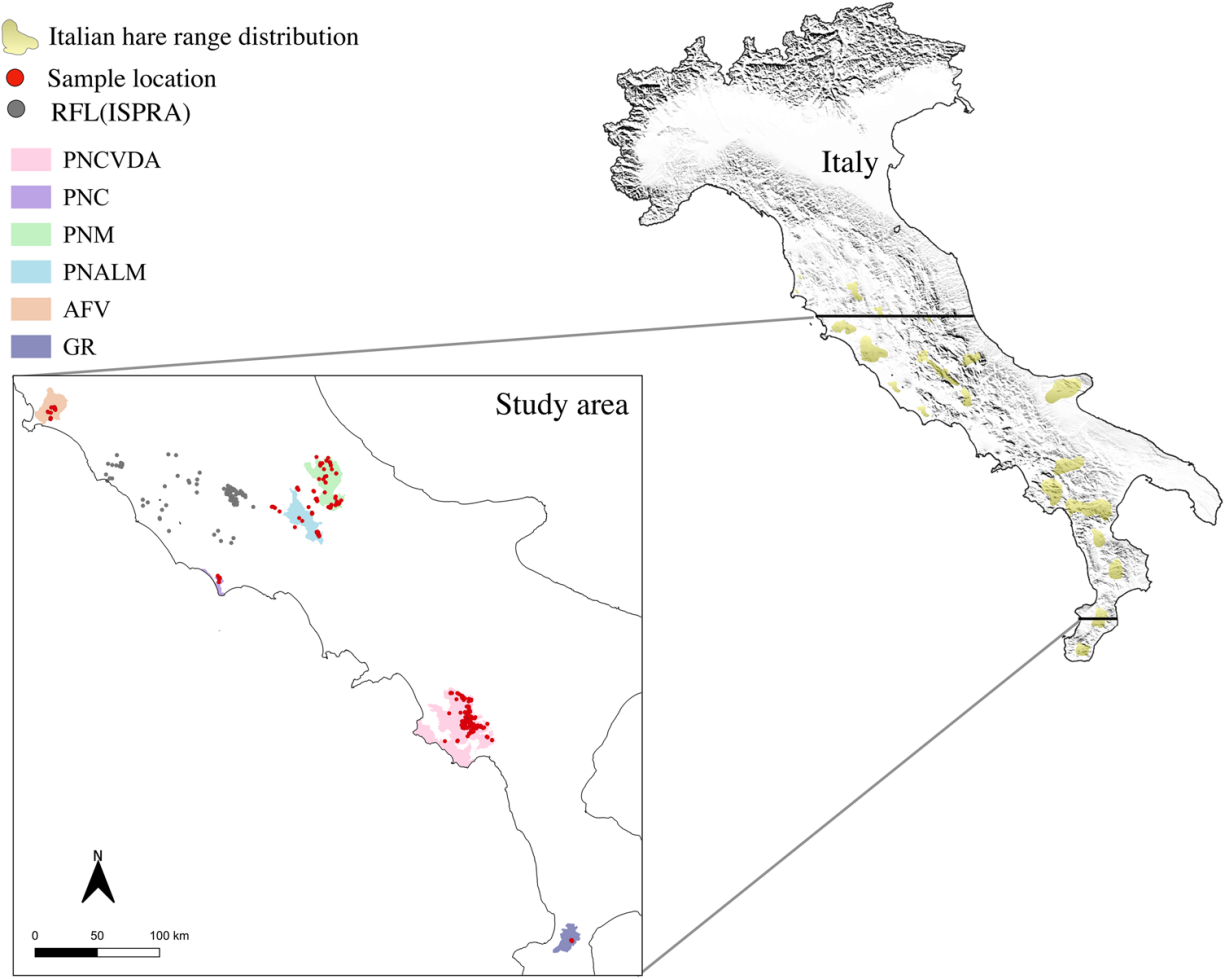
Study area. In yellow, range distribution of the Italian hare in Italy^24,50^, modified). The inset shows the study area with sample locations (red spots, N = 759). *PNCVDA* Cilento, Vallo di Diano e Alburni National Park (in pink), *PNM* Majella National Park (in green), *PNALM* Abruzzo, Lazio e Molise National Park (in azure), *PNC* Circeo National Park (in violet), *AFV* game reserve in Tuscany region (in orange), *GR* Gallo game reserve in Calabria region (in dark purple), *RFL* (*ISPRA*) records on the distribution of the fauna of Latium. Figure was modified from the maps created with software QGIS v.3.4.2^66^, available at https://www.qgis.org.

Here we provide, for the first time, a broad insight on the key traits of the ecological niche of the Italian hare, and its habitat suitability for the whole distribution range of the species on the Italian peninsula (Fig. 1), interpreting the latter after comparing with the same model calculated for the congeneric European hare.

We used a non-invasive sampling (gNIS) approach (see also^27^) on faecal pellets to validate presence data that were employed in the spatial elaboration and the characterization of dietary habits inferred with DNA metabarcoding^28^ and High-Throughput Sequencing (HTS), all together aimed at defining ecological characteristics of the Italian hare.

Information on habitat use^2,18,19,29–32^ and diet^33^ was previously reported, but only for few and local areas, and no investigation before now was available for this species considering such a range distribution.

Our contribution adds important information to broaden the knowledge on the environmental requirements of the Italian hare, representing effective support for fit management actions in conservation planning. Indeed, conservation strategies aiming at minimising habitat loss and inverting wildlife population declines need a clear knowledge of the distribution pattern, abundance and ecological requirements of a species^34,35^ on which to focus management efforts^36^. This becomes particularly important when we consider threatened species, deserving of analyses based on non-invasive approaches and special conservation measures, like the Italian hare.

## Results

Genetic characterization of the faecal DNA reveals that 223 pellets were assigned to the Italian hare and 290 to the European hare. For 107 faecal pellets, High Resolution Melting (HRM)-DNA amplification was unsuccessful (NA = not assigned) and did not allow species assignment (Table 1).

**Table 1:** Summary of samples used in this study.

Source	Total number of samples	LC	LE	NA
PNCVDA (Campania)	391	105	205	81
PNM (Abruzzo)	62	17	37	8
PNALM (Abruzzo)	95	45	37	13
PNC (Latium)	21	21	0	0
AFV (Tuscany)	51	35	11	5
GR (Calabria)	18	12	6	0
RFL (ISPRA) (Latium)	121	45	76	0
Total	759	280	372	107

*PNCVDA* Cilento, Vallo di Diano e Alburni National Park, *PNM* Majella National Park, *PNALM* Abruzzo, Lazio e Molise National Park, *PNC* Circeo National Park, *AFV* Game reserve, *GR* Gallo game reserve, *RFL (ISPRA)* records on the distribution of the fauna of Lazio, *LC* *Lepus corsicanus*, *LE* *Lepus europaeus*, *NA* not assigned.

### Species-specific presence map and potential distribution model

**Figure 2:**
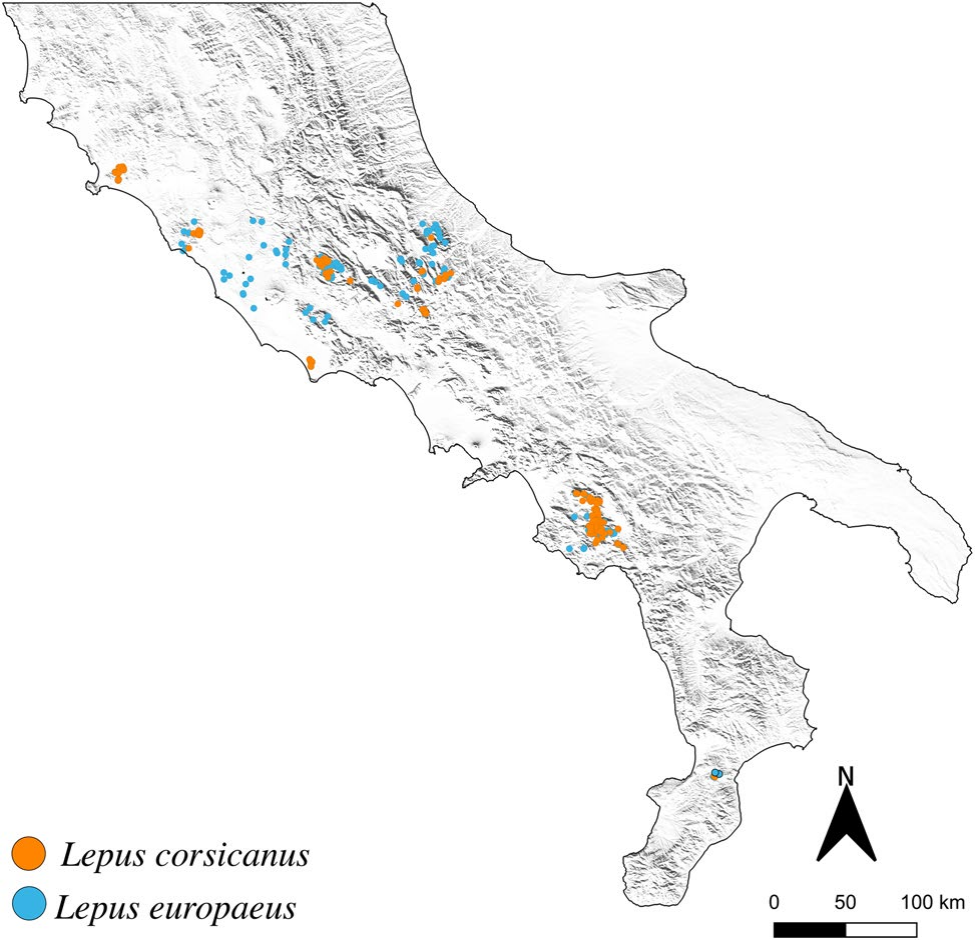
Species-specific distribution map. Distribution map of the samples of the Italian hare (*Lepus corsicanus*, orange spots) and the European hare (*Lepus europaeus*, azure spots). Figure was modified from the map created with software QGIS v.3.4.2^66^, available at https://www.qgis.org.

The spatialization of genetically characterized samples reveals a deep sympatry between the Italian hare and the European hare in the study area (Fig. 2). For the maximum entropy (MaxEnt) distribution model, 280 presence locations for the Italian hare and 372 presence locations for the European hare were both used. After removing replicated geographical coordinates, 234 and 261 presence locations of the Italian hare and the European hare, respectively, were retained for the analyses.

**Figure 3:**
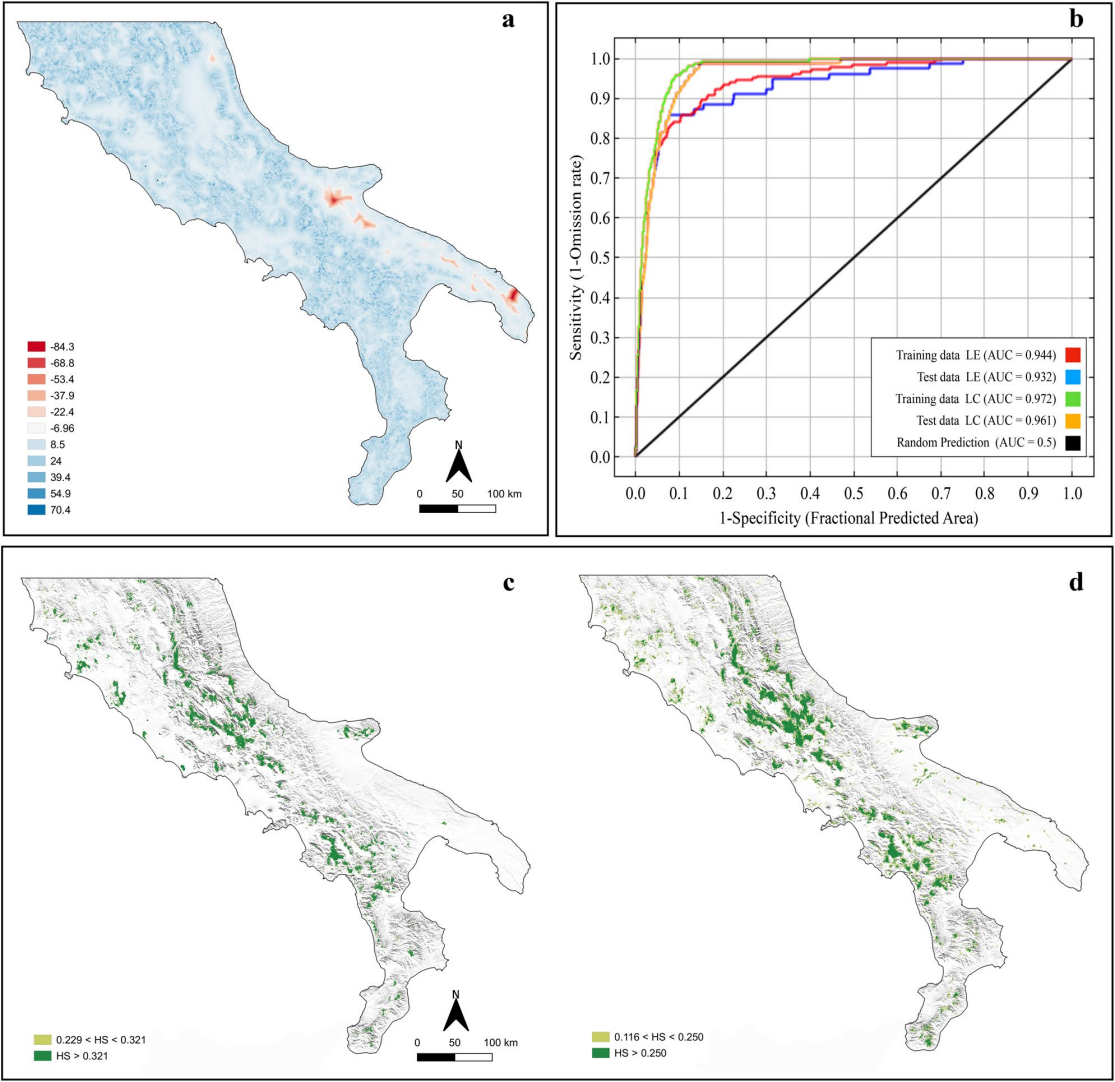
Habitat suitability analysis for the Italian hare and the European hare. (**a**) Multivariate environmental similarity surface (MESS) showing areas where model predictions are extrapolations in comparison to the training data set. The red areas show one or more environmental variables outside the range. (**b**) Receiver operating characteristic (ROC) curves for the Italian hare (LC) and the European hare (LE) of the models, AUC area under the curve. Habitat suitability maps of (**c**) the Italian hare (LC) and (**d**) the European hare (LE) based on MaxEnt models using 13 environmental variables and 234 and 261 presence points, respectively. 10 percentile training presence (0.321 for LC and 0.116 for LE) and Maximum training sensitivity plus specificity (0.229 for LC and 0.250 for LE) were used as thresholds for probability of presence (HS). Figures were modified from the maps created with software QGIS v.3.4.2^66^, available at https://www.qgis.org.

The multivariate environmental similarities surface (MESS) analysis reveals how reliable the prediction is outside the sampling region. In the MESS map (Fig. 3a), areas in red have environmental variables outside the range present in the training data, among these are included distance from natural and seminatural grasslands, deciduous woods, rivers and scrub. For most of the Apulia region, our model is less reliable than in the rest of the peninsula.

The average test AUC (the area under the receiver operating characteristic curve^37^) for the replicate runs was 0.960 with a standard deviation of 0.006 for the Italian hare, and 0.915 with a standard deviation of 0.020 for the European hare. The test AUC for the final models were 0.961 and 0.932 for the Italian hare and the European hare, respectively (Fig. 3b), which denotes high performance of the predictive model^38^.

The thresholds considered for habitat suitability were the 10 percentile training presence^39^, which resulted in values of 0.321 for the Italian hare and 0.116 for the European hare. The maximum training sensitivity plus specificity^40^ resulted in values of 0.229 for the Italian hare and 0.250 for the European hare.

The habitat suitability map shows that the potential distribution of the Italian hare (Fig. 3c) expands mainly to the Central-Southern mountain region, in addition to some coastal spots in the Northern-Central Tyrrhenian portion, with a total area of the highest probability of presence of 7,688.28 km^2^. This range seems to be characterised by more continuity in the Central part of the peninsula (i.e. Majella National Park, PNM areas and Abruzzo, Lazio and Molise National Park, PNALM, areas), becoming more fragmented going towards the South of Italy and coasts. In particular, isolated patches of suitable habitat are more evident in sub-coastal areas of Viterbo (Lazio), Tyrrhenian coast, and internal mountain regions of the southern portion of the distribution range. On the Eastern coast of Italy, territories with suitable environmental characteristics for the Italian hare are localised on the Gargano promontory areas (North Apulia).

The habitat suitability analysis for the European hare (Fig. 3d) shows a potential distribution pattern near the Apennine ridge similarly to the Italian hare, albeit with a clear greater extension (14,713.8 km^2^). Along the Apennines, the patches, potentially suitable to the European hare, are better connected to each other and the coastal plains are more interested. Indeed, many other potential distribution sites, that exclude suitable habitat characteristics for the Italian hare, can be highlighted, such as some Tyrrhenian costal line areas of Campania, Mount Vesuvius, and some internal areas of Apulia Calabria, and Basilicata. These areas account for 7% of the total peninsular territory considered.

A spatial overlap with high suitability for the Italian hare and the European hare exists for many local areas, reaching the value of 84.6% of total suitable habitat for the Italian hare.

Analyses of variable contributions shows that the most relevant environmental variables affecting habitat suitability for the Italian hare are distance from tree plantations (25.7%), urban elements (15.1%), agricultural meadows (14.1%), deciduous woods (11.9%), as well as elevation (10.4%) (Table 2).

**Table 2:** Estimates of relative contributions of the environmental variables to the Maxent model for the Italian hare (LC) and the European hare (LE).

Variable	Percent contribution	
	LC	LE
Tree plantations	25.7	13.9
Urban elements	15.1	2.3
Agricultural meadows	14.1	28.9
Deciduous wood	11.9	3.7
Elevation	10.4	24.6
Natural and seminatural grasslands	7.8	9.4
Slope	2.8	3.2
Mixed woods	2.8	5.9
Waterways	2.6	1.7
Roads	2.6	3.1
Coniferous woods	2.2	0.4
Scrub	1.1	1.2
Aspect	1	1.7

For details see also Supplementary Fig. S2.

The first five environmental variables affecting model of distribution for the European hare are distance from agricultural meadows (28.9%), elevation (24.6%), tree plantations (13.9%), natural and seminatural grasslands (9.4%), and mixed woods (5.9%). The way in which these variables affect the distribution model of the two considered species, varies both among variables and according to the size of the same variable (Supplementary Fig. S2). For example, distance from tree plantations, in both species, rises the probability of presence at first and then decreases when the size of the variable increases. On the other hand, as the distance from urban elements increases, the probability of the presence of the two hares also increases (Supplementary Fig. S2).

Some authors reported elevation affecting the distribution of hares in some local populations^22^. Accordingly, elevation in the present broader analysis was one of the environmental variables contributing to the definition of the suitable habitat both for the Italian hare (10.4%) and for the European hare (24.6%) (Table 2).

**Figure 4:**
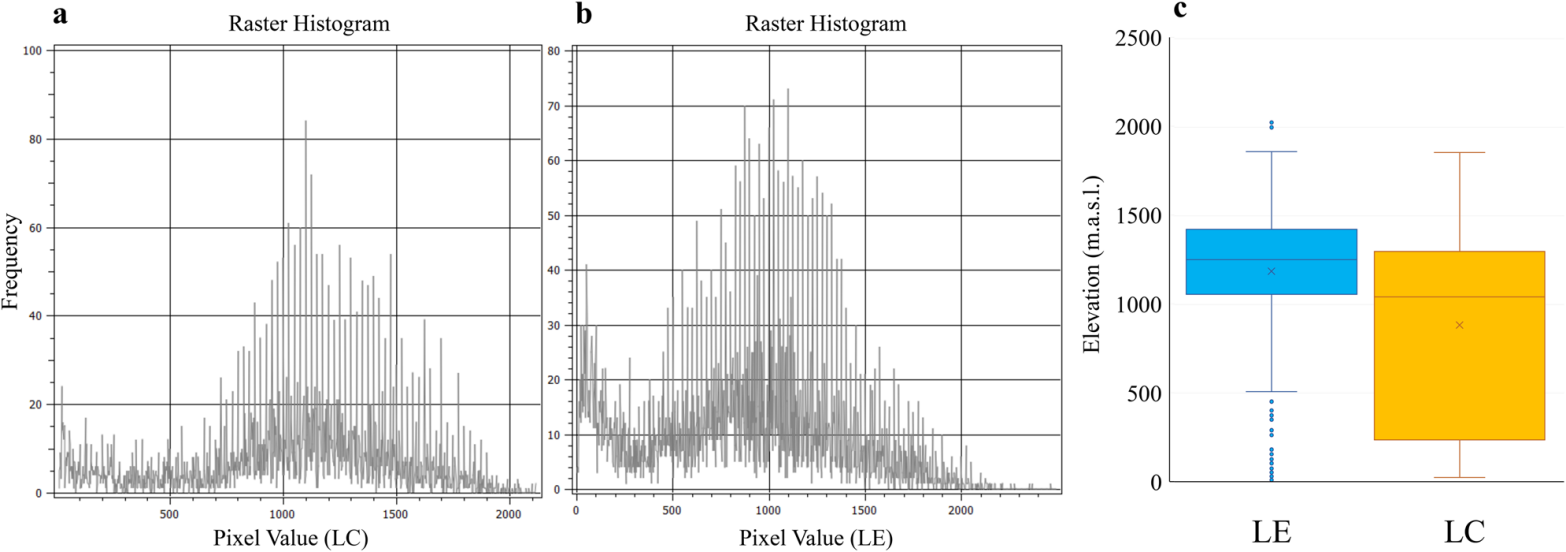
The distribution of hares related to elevation. Altitudinal distribution of the suitable habitat for (**a**) the Italian hare and (**b**) the European hare; (**c**) observed distribution of the Italian hare (LC) and the European hare (LE); *m s.a.l.* meters above sea level.

Taking in mind the 10 percentile training presence threshold, the potential altitude distribution of the Italian hare ranges from 4 to 2,139 m a.s.l., with frequency higher than 50% between 1,000 and 1,500 m a.s.l, and the highest peak (85% frequency) at 1,100 (Fig. 4a). Despite this relevance, seasonal variations in the occupancy of different altitudinal ranges scale were not detected. Indeed, the boxplot plotted performed using all the location points for of the Italian hare (from the present survey), shows that both Spring/Summer (SS, from March to July) and Autumn/Winter (AW, from September to February) samples are spread, on average, at to 981 and 1,018 m.a.s.l., respectively (Supplementary Fig. S3), nevertheless, no statistically significant difference was found between two seasons (one way ANOVA test, *p* value > 0.05).

The European hare shows a potential altitude distribution ranging from 1 and 2,496 m a.s.l., with a frequency higher than 50% between 750 and 1,350 m a.s.l., and the highest peak (about 72% frequency) at 1,100 m a.s.l. (Fig. 4b).

The comparison of the observed altitude distribution (Fig. 4c) shows a significant difference (one way ANOVA test, *p* value <  < 0.01) between two species. The Italian hare samples spread on average at 884.323 m a.s.l., while samples of the European hare show and average distribution at 1,186.89 m a.s.l. Furthermore, starting from low altitude for both the Italian hare (minimum: 23.5 m a.s.l.) and the European hare (minimum: 10.20 m a.s.l.), the latter reach higher elevation (maximum: 2,034.28 m a.s.l of the European hare vs 1,856.36 m a.s.l of the Italian hare).

### Diet of the Italian hare

Foraging opportunities are often observed to affect range size and spatial distribution^41^. For diet analysis of the Italian hare we used faecal DNA (N = 101) showing the best quality parameters (> [50 ng/μL]; λ_260/280_ > 1.60; λ_260/230_ > 1.60) and collected homogeneously in all areas of sampling (Fig. 1).

A total of 14,854,138 short raw reads (35–301 bp) were obtained from Illumina MiSeq sequencing. The reads were separately processed and filtered, and 1,071,347 sequences resulted after the bioinformatics analysis of the data. Of these sequences, the *blast* against NCBI (National Center for Biotechnology Information) database allowed the taxonomic assignment at a resolution of 49.52% (N = 28) for family level, of 40.78% (N = 111) for genus level, and 9.69% (N = 196) for species level. During the sequencing of controls, no contamination was detected.

**Figure 5:**
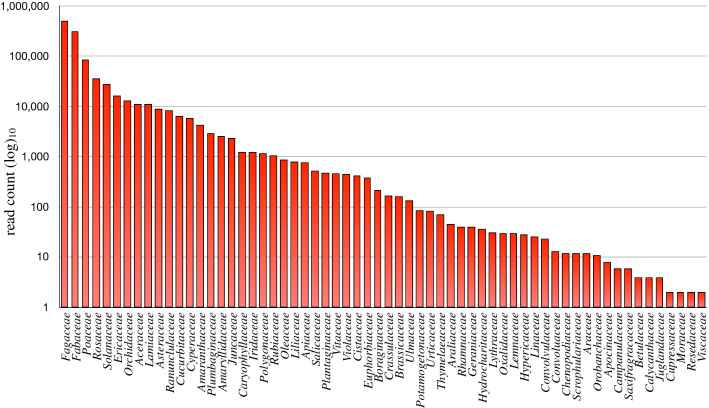
Diet of the Italian hare all year-round. Number of read count (log_10_) of each plant family included in diet of the Italian hare all year round.

The year-round diet of the Italian hare includes 344 plant taxa belonging to 62 families and 192 genera. The *Fagaceae*, *Fabaceae*, *Poaceae*, *Rosaceae* and *Solanaceae* (counts > 20,000) emerged as the most frequently occurring families, accounting for 90.22% of the total diet. All the other families show a frequency of occurrence < 2%) (Fig. 5 and Supplementary Table S1). *Poaceae* (74 items), *Fabaceae* (38 items), *Asteraceae* (24 items), *Rosaceae* (21 items), and *Orchideaceae* (10 items) are the families showing the greatest richness, meaning the number of plant items (Supplementary Table S1). In particular, *Onobrychis sp.* genus represents 21.76% of the total items ingested by the Italian hare and the 74.51% of all *Fabaceae.*

**Figure 6:**
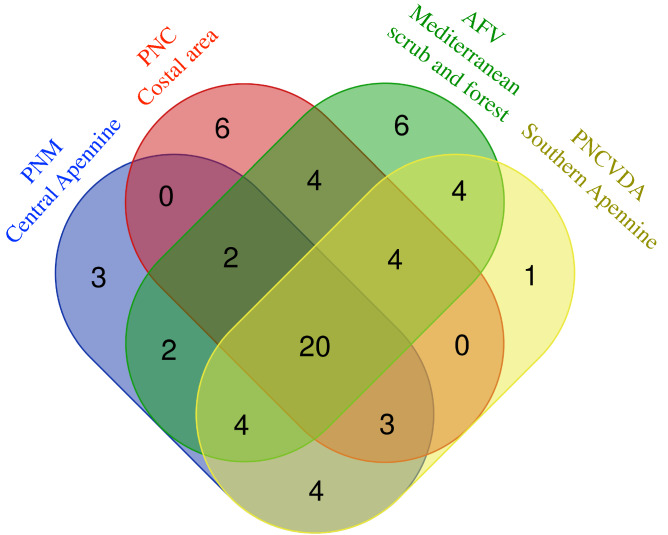
Symmetric Venn diagram of shared and unique plant families in diet of samples collected in different environmental typologies. Cilento, Vallo di Diano e Alburni National Park (PNCVDA), Majella National Park (PNM), Circeo National Park (PNC) and Game reserve (AFV), produced by Venn Diagram Tool freely available on the web (https://bioinformatics.psb.ugent.be/webtools/Venn/). See Supplementary Table S2 for details.

Although we aimed to define the food habits of the Italian hare in terms of plant items occurring in the diet, our extensive survey allowed to highlight an intraspecific variability in the diets of the Italian hare populations coming from our sampling areas (Fig. 6 and Supplementary Table S2), that show different geographical and environmental characteristics. The Italian hare populations living in the Central Apennine (i.e. PNM and PNALM areas) share 4 plant families with those from Southern Apennine mountains (i.e. Cilento, Vallo di Diano e Alburni National Park, PNCVDA, areas), 2 plant families are shared with populations living in Tuscan hilly areas (i.e. an hunting reserve managed by Wildlife Sector of the Tuscan Regional Council, AFV) and none with individuals from the Circeo (Circeo National Park, PNC) coastal areas (Fig. 6 and Supplementary Table S2). The latter show diets including no plant families shared with hare population from Southern Apennine but 4 plant families occurring also in diet of samples from Tuscan hilly areas (Fig. 6 and Supplementary Table S2). The Italian hare diet shows 20 plant families that are shared between all sampling areas (Fig. 6 and Supplementary Table S2).

The SS and the AW seasonal diets are characterised by 60 and 49 families, respectively, sharing 47 plant families (Supplementary Table S3). Although these diets exhibits some differences (Supplementary Fig. S4) depending on the availability of trophic resources during the season, it may highlight the prevalence of one plant family: *Fabaceae* (60.70%) in SS diet, and *Fagaceae* (67.47%) in AW diet, followed by other 57 and 48 families with relative frequency of occurrence < 10%, for the SS and the AW diet, respectively (Supplementary Table S1).

When the same sequence was assigned to more than one taxon, the higher taxonomic level that included all of these, was selected. This hierarchical assignment allowed diet characterisation of samples at different taxonomic level (family, genus and species). *Onobrychis* sp. is the most representative genus both in the SS diet (43.59%) and the AW diet (8.41%), although in the latter it is the *Fagaceae* family that explains 60.01% of the total diet in autumn/winter season (Supplementary Fig. S4 and Supplementary Table S1).

Considering all plant items, the SS diet shows a higher richness (N = 266) than the AW one (N = 199), according to α-diversity calculated with Shannon index, that displays a value of 2.329 and of 1.818 for the SS and the AW diet, respectively (Table 3; Kruskal–Wallis test *p* <  < 0.01).

**Table 3:** Diversity index for spring/summer (SS) and autumn/winter (AW) diets of the Italian hare.

	SS	AW
Richness	266	199
Shannon	2.328	1.818
Evenness	0.03858	0.03096
Equitability	0.4169	0.3435

## Discussion

Spatial and feeding ecology are closely related to each other, and together they can contribute to the definition of the ecological niche of a species. This detailed knowledge in terms of the animal’s requirements, sometimes becomes difficult to obtain due to the elusive habits or vulnerable conservation status of a considered species. Indirect information, mainly from non-invasive samples, allow us to get data (i.e. genetic profile, presence, distribution, habitat preferences, food requirements)^42^ for ecological surveys, without any contact with animals or minimising the disturbance to them. Indeed, the use of gNIS provides practical and ethical method, preferable to other strategies to study animal ecology, that manipulate the individuals, inducing stress and mortality, or require high field effort, such as live-trapping. This is particularly useful when working on elusive species, like hares, or rare and vulnerable species like the endemic Italian hare^25,27^. However, faecal DNA is often highly degraded^43^, it contains low template concentration and it carries PCR inhibitors from the environment or the digestive system, reducing the success of the genetic analyses^44,45^.

Here, we depicted some features of the ecological niche of the Italian hare using the population of Central-Southern Italy, investigating on over 60% of the peninsular populations.

The wide field experience combined with a laboratory approach showed that only the freshest faecal pellets minimise some of the limitations linked to the use of faecal DNA and increase the power of the information provided by molecular analyses. Moreover, we also implemented some measures to remove inhibitors, like the incubation of the faecal materials with InhibitEX buffer (Qiagen, Hilden, Germany) during the extraction of DNA used in HRM for species characterization. These efforts provided more reliable genetic data that we used to infer about the distribution and ecological preferences for the Italian hare.

Our analyses showed that the species distribution depends on eco-geographical variables of woodland and agricultural environments, affected by altitude and urban elements. In particular, the probability of meeting the Italian hare is positively correlated with the distance from the woods and agricultural environment up to a threshold, beyond which there is a decrease, suggesting that its optimum resides in ecotonal environments. The human environment, otherwise, always seems to be negatively correlated, confirming the intolerance of the Italian hare for human presence and strongly corrupt environments^19,29,31,46^. The European hare could represent an element of exclusion from the environments preferred by the Italian hare, if we consider that at least 4 of the first 5 heaviest variables are shared between the two species.

In addition, for this competitor of the Italian hare, a greater adaptability is clear, as can be seen from broader and higher values of the curves that describe the probability of presence (Supplementary Fig. S2).

The observed altitudinal distribution showed that there was no significant difference between the Spring/Summer (SS) and Autumn/Winter (AW) seasons in the preferred distribution of the Italian hare. Locations of all collected samples taken together show an altitude distribution range spanning from 0 to 1,850 m a.s.l., a lower range than the more widely distributed European hare (which reaches 2,000 m).The range from sea level to 100 m a.s.l. of the Italian hare includes the faecal pellets collected in the Circeo areas and in Province of Grosseto (Tuscany) areas, as well as the occurrences from Lazio (RFL, that push the altitudinal range down). In the sub-interval from 100 and 500 m a.s.l., the Italian hare and its suitable habitats seem to disappear, while the European hare shows higher frequency values. A plausible interpretation of this pattern could ascribe to the disappearance of the Italian hare from low altitudes as a result of human pressure and habitat alterations (i.e. hunting, poaching and urbanisation)^2^.

Some studies reported the Italian hare historically widespread in different environmental typologies (i.e. woods, grasslands, scrubs) and altitudinal ranges (i.e. plain, hill, mountain) with morphological traits adapted for Mediterranean climates [^2,47^, Angelici, 1970 personal comment]. In addition, until 1930, the species was reported also on the isle of Elba, subsequently extinct^48^. All recent attempts to reintroduce the Italian hare on the island have proved difficult to realize, due to excessive anthropic disturbance^30^.

Nowadays, the Italian hare is described as an occupant of mountain pastures, woods and ecotone, surviving at low altitude and close to coastline only in few local areas in Italy, such as in Latium^49^ and in the Province of Grosseto (Tuscany), as well as in Corsica^50^.

In this scenario, the populations from coastal areas could be considered as a relict population, probably due to the preservation of which some of them have benefited, as a result of living in a protected area.

Learning more about the food choices of the Italian hare could be useful in interpreting other dimensions of the niche of this species. Feeding habits, referred to an extensive survey, confirm and broaden previous studies performed for few local areas in Central^29,31^ and Southern Italy^19^.

The diet of the Italian hare was defined by using DNA metabarcoding^28^ and HTS, that is considered one of the most accurate strategies available for the understanding of the food habits of animals^43^, outdoing some of the limits of the morphological methods^51–53^. In fact, the latter (i.e. microhistological identification of undigested plants fragments in faecal pellets) depends from operator skills^53^ and are sometimes only useful considering solid remains that retain diagnostic taxonomic features^54^. Furthermore, morphological keys could be lost or altered during digestion or lagomorph caecotrophy^55,56^, leading to non-exhaustive and low-resolution analysis.

The diet of the Italian hare shows a wide spectrum, encompassing 334 plant taxa belonging to 62 families and including both herbaceous and arboreal (i.e. trees and shrubs) items (Supplementary Table S1). Our analysis should mirror most of the real plant components of the diet. This should be true if we consider previous diet molecular study^24^ showing that the number of plant families, based on 22 faecal samples collected in local areas of Central-Southern Italy, represented only 48.0% of the total plant families revealed in our wider survey.

*Fagaceae*, *Fabaceae*, *Poaceae* and *Rosaceae* were the most abundant families in the diet all year round (Fig. 5). In particular, *Fagaceae* (probably barks and buds of mature beeches and oaks) and *Rosaceae* (probably edible fruits and roses) were ingested in high percentage during the Autumn/Winter season, when plants of the sward are characterised by a vegetative phase such as to satisfy the requirements of the species. Similarly, *Fabaceae* (spontaneous and cultivated plants: leguminous forbs and cereals) and *Poaceae* (grasses) were the most frequent items ingested during the Spring/Summer season suggesting foraging behaviour on *Onobrychis* sp. grasslands and *Trifolium* sp. pastures.

These findings agree with habitat preferences of the Italian hare for woodland and cultivation during cold and warm seasons, respectively^31^.

In addition to dominant *Fagaceae* in the AW season and *Fabaceae* in the SS season, other 48 and 59 families respectively, were included in the seasonal diet of the Italian hare (Supplementary Table S1 and Supplementary Fig. S4). This could suggest that the Italian hare utilises the food resources according to their availability, preferring the most abundant ones^57^. For example, *Fabaceae* and *Poaceae*, although were ingested at high frequency both during the SS and the AW seasons (Supplementary Table S1), were consumed further during their full flowering phase^33,58,59^. Furthermore, the SS diet showed higher richness than the AW one (Table 3), probably according to higher plant availability in this season.

Also, diversity indices elaborated according to the SS and the AW diets (Table 3), showed variation in food choices, with all values consistently higher for the SS than the AW. This difference could depend on dominance showed by just one taxon in the AW diet followed by others at low frequency of occurrence. For example, *Fagaceae* explains 67.47% of the total AW diet while all other plant families each show up with < 10%. This result could be correlated to seasonal availability of food items, although plant seedlings are high in all seasons.

The SS and AW diets, despite this significant difference in diversity indices, shared 47 plant families. This may suggest that food habits of the Italian hare are not only affected by seasonal variation, as a consequence of a generalist food behavior of the species^24,33^. It would be interesting to investigate seasonal plant availability to confirm this hypothesis. Probably, some plant items represent the “hard core” of the diet and these elements are the variables affecting the selection of the habitat by the Italian hare.

## Conclusion

The Italian hare occurs preferentially in hilly and mountain habitats characterised by high environmental heterogeneity such as meadow pastures (also grazed by domestic and other wild animals or human managed), forests with ecotones and avoid human corrupted habitat. To date, the Italian Apennine mountain systems still retains many environmental typologies that appear suitable for the ecological requirements of the Italian hare, and this could represent an opportunity for the management of this currently vulnerable species.

Our analyses suggest that the Italian hare seems to be an adaptable species, able to exploit a wide range of ecological resources in different environmental contexts and, often, in sympatry with populations of the European hare. Taking these considerations into account, we speculate that the threats to the status of the species are probably not linked to the lack of habitat, but more likely to ecological competition combined with anthropogenic landscape fragmentation^1,5,19^.

Assuming this hypothesis, we provide helpful ecological data that increase knowledge useful to management actions aimed at safeguarding the Italian hare. These efforts could be directed to the habitat typology preferred by the species and housing the plant elements included in its diet. Some studies reported that long-term survival of populations of vertebrates could be achieved by protecting the source populations and providing dispersal opportunities by connecting them^60–63^.

For example, starting from suitable habitats for the species, these plans could provide releases of captive animals to contain the effects caused by low density population and/or for designing ecological corridors aimed to increase the link between isolated populations. Furthermore, next steps could be directed at deepening the interaction between the Italian hare and European hare to detail their possible ecological competition, including this information in the definition of management actions. Indeed, since recent hybridization between the Italian hare and the European hare is not reported^26^, the two species could interact directly or indirectly, depending on trophic resources competition. Considering this, a better knowledge of the European hare diet (via DNA metabarcoding) and a home range analysis in sympatry areas of the two species could be advantageous to achieve a proper management and conservation plan for the endemic species.

## Methods

### Study area

The study was carried out in Central-Southern Italy (Fig. 1), including different geographical localities that could be considered representative of the entire distribution range of the Italian hare in the peninsula^24,50^.

According to the National Project for the conservation of the Italian hare (Progetto di Sistema, Conservazione della lepre italica) funded by the Ministry for Environment, Land and Sea Protection of Italy, sampling was performed within and around protected areas:

- PNCVDA, 40°17ʹN, 15°19ʹE—Salerno, Campania;
- PNM, 42°4′N, 14°3′E—Sulmona, Abruzzo;
- PNALM, 41°48′N, 13°47′E—Pescasseroli, Abruzzo;
- PNC, 41°14′N, 13°3′E—Latina, Latium;
- AFV, 42°31′N, 11°22′E—Grosseto, Tuscany;
- GR, 38°43ʹN, 16°22.680′E—Cenadi, Calabria.

Faecal pellet collection was performed in different environmental typologies with various morphological and vegetational characteristics, mirroring the diverse landscapes of the Italian peninsula. Most of the samples were collected in the Apennine mountain system (i.e. in PNM, PNALM and PNCVDA) with steep slopes covered with forests and meadows and pastures occurring on the plateau and gentler slopes. The mountains were dominated by forests of *Fagus sylvatica* interspersed with secondary origin grasslands and clearings composed mainly by *Poaceae*, *Fabaceae* and *Cyperaceae*. *Alnus* sp. and *Castanea* sp. forests are found in the underlying altitudinal range, followed in the medium-lower altitude level by deciduous oaks and maple trees*.* Xeric grasslands of meadow brome are the most common.

Lowland forests are frequent on the coastal line (i.e. in PNC), alternated with dry areas dominated by *Quercus* sp. formation and luxuriant Mediterranean scrub. The latter is enriched with sclerophilous forest composed by *Juniperus oxycedru*s, *Myrtus communis*, lianose plants, like *Clematis vitalba* and *Smilax aspera*, and *Arbutus unedo*. The bush is dense and complex, dominated mainly by *Ruscus aculeatus*, and *Asparagus acutifolius*. Arboreal level consists of *Quercus* sp., *Alnus* sp. and *Populus* sp. together with hygrophilous herbaceous plants. Promontory areas include high bush, typical Mediterranean evergreen forest dominated by holm oak, *Arbutus unedo, Spartium junceum,* xerical vegetation and cultivated areas (i.e. in AFV and on hilly areas of the Tuscany region and GR in Calabria). The cliffs dress with bushes of *Euforbia dendroides*. Finally, rivers, lakes and channels are dominated by swamp and salty vegetation, mainly characterised by *Amaranthaceae.*

### Samples collection

From 2015 to 2019, we conducted an extensive field survey to record the presence of the Italian hare and collect faecal pellets that were used for laboratory procedures (PNCVDA N = 391; PNM N = 62; PNALM N = 95; PNC N = 21; AFV N = 51). The sampling was performed using visual searching by field collectors and/or scat detection dogs, trained by positive reinforcement for hare scats recognition^64^, within altitudes ranging from 0 to 2,000 m a.s.l.

Only fresh scats (0–2 day old), aged by skilled field collectors, were gathered (Supplementary Fig. S1).

The samples were handled in sterilized conditions and preserved in sterile tubes with silica desiccant granules^65^, stored at 4 °C (during transport) or − 20 °C (in laboratory).

All records were geo-referenced using a global positioning system and loaded in GIS environment using the software QGIS 3.4.2^66^ (https://www.qgis.org).

### High resolution melting analysis to species assignment

The sympatry between the Italian hare and the European hare, and the difficulty in discriminating the species using aspect pattern of faecal pellets, made species assignment necessary.

DNA was extracted from the external surface of each faecal pellet with the QIAamp DNA Fast Stool Mini Kit (QIAGEN GmbH Valencia, CA, USA) according to guidelines. To exclude potential cross-contaminations, blank extractions were included. DNA quality and quantity were checked using gel electrophoresis run, Nanodrop ND-2000 (Nanodrop, Wilmington, DE, USA) and Qubit Fluorometer 3.0 (Thermo Fisher Scientific).

Species assignment was developed using HRM analysis^67^ on Rotor-Gene Q 5-Plex (QIAGEN GmbH Valencia, CA, USA) with the Type-it HRM PCR Kit (QIAGEN GmbH Valencia, CA, USA). Experimental protocol and raw data analyses (using Rotor-Gene Q software v. 2.1.0 (QIAGEN GmbH Valencia, CA, USA) were performed following^25^. The assignment of unknown samples to corresponding hare species was made using their resulting melting profile compared to positive controls melting curves.

### Distribution and habitat suitability model

The potential distribution for the Italian hare, as well as for the European hare, was estimated using geographical points associated to samples genetically assigned to this species. This data was integrated with records of certain Italian hare and European hare specimens from Lazio (RFL) (collected between 2007–2008), freely downloaded from Istituto Superiore per la Protezione e la Ricerca Ambientale (ISPRA) online database (https://geoviewer.nnb.isprambiente.it/) and samples from Gallo reserve, a hunting game reserve in Calabria (collected between 2015–2018).

In the spatial elaboration, we included the entire distribution range of the Italian hare on the peninsula^2^, excluding Sicily, considering that the Sicilian population of the Italian hare shows genetic differences^10^ and ecological divergence^15^ compared to populations living on peninsula. Furthermore, despite the (unsuccessful) release of the European hare in recent decades^18^, the Italian hare is the only hare species present in Sicily^68^, where its occurrence is continuous^1^, making the Sicilian population not subject to serious threats^69^.

The correlation between species presence and its biophysical environment was made using MaxEnt distribution model^70,71^ in MaxEnt version 3.4.1 (https://www.cs.princeton.edu/). MaxEnt is widely used for conservation biology^72–74^. It was considered the best among many other species distribution models^75^, mainly because it requires only presence data and the predictor variables, using continuous and categorical data at a single time^39,76,77^.

A total of 13 spatially explicit variables (SEV) were selected, based on their potential importance, inferred by our knowledge and published sources^19,22,29^. These variables included: (a) topographical variables, such as altitude, slope, and aspect,(b) hydrological, such as waterways,(c) anthropogenic comprising buildings and roads; and (d) vegetational, such as deciduous, coniferous, and mixed woodlands, natural and seminatural grasslands, agricultural meadows, Mediterranean scrub, tree plantations, as categorised in Corine Land Cover land use (maps from 2018, level 3, scale 1:50.000).

Altitude was determined using a Digital Terrain Model (DTM) resampled at 100 m (https://www.pcn.minambiente.it), aspect and slope were calculated using the GDAL functions “Aspect” and “Slope” in QGIS 3.4.2^66^ (https://www.qgis.org), respectively. The remaining 10 variables were obtained as shapefiles (https://www.pcn.minambiente.it for waterways and roads; https://land.copernicus.eu/ for the Corine Land Cover files) and rendered as continuous rasters by considering the Euclidean distance from each feature, using the GDAL function Proximity (raster distance), and selecting geographical units. Spatial elaboration of the SEV maps was done in QGIS 3.4.2^66^ (https://www.qgis.org). All SEVs were rendered as raster maps with a resolution of 100 × 100 m.

In order to avoid collinearity between the predictor variables, Pearson’s correlation coefficient was calculated, to ensure none of the variables showed a coefficient higher than 0.70^78^. For both the Italian hare and the European hare, the training areas used were the regions that included our sampling areas, and the model was then projected to the rest of peninsular Italy.

Models were generated using default settings, with a random test percentage set at 30% and removing duplicate coordinates. A regularization parameter of 1.0 was used, the maximum number of iterations was set at 500 and a jackknife procedure was used.

Performance was estimated for each model by calculating the average test AUC^37^ through fivefold cross‐validation. The AUC value, that is among the statistics most used in distribution modelling of species^70,79^, is calculated by measuring the area under the receiver operating characteristic (ROC) curve. This value ranges from 0 to 1, where AUC score of 1 represents a perfect model. Following this, the model was run a final time for each species, with the same settings, and these final models were used for subsequent analyses.

To reveal how reliable the prediction is outside the sampling region, we performed the MESS analysis with which we compared the environmental similarity of variables in the Italian peninsula to the environmental data used for training the model^80^.

### Diet characterization

For diet characterisation we used DNA metabarcoding coupled with HTS on the faecal pellets.

#### DNA isolation and amplification

DNA was extracted, in a dedicated room, with the hexadecyltrimethylammonium bromide (CTAB) method^81^, starting from the internal material of faecal pellets. The PCRs were performed as described in^24^, using the JK11 (5′- ATCCTGGAATTCACAACCAAGTATCG -3′) and JK14 (5′- GGAGAAGTCGTAACAAGGTTTCCG -3′) primers^82–84^ that allow the amplification of 350 bp of nuclear Internal Transcribed Spacer 1 region. The amplification primers were modified adding a specific adapter to their 5ʹends, in order to retrieve sequences from each sample post-sequencing^85^. Two PCR replicates were performed for each sample. Then, PCR products were purified by Illustra GFX PCR DNA and Gel Band Purification Kit (GE Healthcare, Buckinghamshire, UK). PCR replicates for each sample were combined, and DNA in all PCR samples were mixed in equimolar concentrations^86^.

A minimum of four faecal pellets were used to obtain a single pool that was tagged for a corresponding library^24^. All faecal DNAs useful for molecular diet characterization were grouped in 8 pools according to AW (N = 49) season and eight pools according to SS (N = 52) season.

#### Sequencing and data analyses

Large-scale sequencing was performed with a 2 × 300 bp paired-end run using the Illumina MiSeq platform (Illumina, Inc., San Diego, CA, USA), well-known to produce lower error rates than other next generation platforms^87^. The Nextera DNA Sample Preparation protocol was used for sequencing by the Genomix4Life Srl (https://www.genomix4life.com/it/). Separated pre- and post-amplification areas were designated for library preparation to avoid any contamination. Sequencing of a sample containing a known microorganism was used as internal positive and negative control to check for contamination during the preparation of the sequencing libraries. Finally, the library was tested using TapeStation (Agilent Technologies, Santa Clara, CA).

Quality control of raw reads and diet data analyses was conducted as described in^24^. In particular, the quality check on raw reads was performed by the FastQC software v0.11.4 (https://www.bioinformatics.babraham.ac.uk). The clipping of the primers and Illumina adapters, and trimming of the low-quality raw reads (Q < 28) were carried out by the Trimmomatic software v0.35^88^. Paired reads were processed, removing orphan reads and keeping only resulted filtered reads for subsequent analysis. The SOAPdenovo2 127-mer^89^ software was implemented for assembly, making overlapping and contiguous sequences (contigs).

To assign each sequence to the corresponding taxonomic level, the contigs sequences were identified by querying against all nucleotide records in NCBI using the BLAST 2.3.0 + software^90^. Only the alignments with an E-value < 0.1 and a ratio between the length of the sequence and the alignment (alignment score) > 50% were selected. Biogeographic information and a regional list of plant species (http://www.actaplantarum.org) were used for increasing the accuracy of the automatic taxonomic assignation, in order to reduce the common misassignment of a sequencing contig to the operational taxonomic units^91^. If the same contig was assigned to two or more different taxa, we selected the higher taxonomic level that included all of these. Sequences assigned to a taxonomic rank higher than family were labeled as *unclassified* and were discarded from the subsequent analyses. Mapping for quantitative analysis was performed using the bwa-0.7.12^92^, samtools 1.3^93^ and samstat 1.5.1^94^ software. The reads were aligned with the reference contigs, previously assigned to the corresponded taxa during the *blast*. The number of reads was calculated for each taxonomic assignment, removing sequence with reads count = 1^95^.

Reads count were summed up to obtain the diet of the Italian hare all year round and seasonally (AW and SS).

### Statistical analysis

The results were expressed as frequencies of occurrence (%) of each plant item (species, genus, family level) in the faecal pellets, both annually and seasonally. Frequency of occurrence (%) is defined as the number of the HTS filtered reads assigned to a taxon, divided by the total number of reads of all identified plant taxa.

Using Past v. 3.2. software^96^, we, firstly, elaborated alfa diversity descriptors (Shannon index, Evenness index, Equitability) for the SS and AW diets of the Italian hare,then, to assess the normality of distribution of our data, we used a Shapiro–Wilks test^97^,finally, we performed a Kruskal–Wallis test^98^ considering all plant items in the SS and AW diets of the Italian hare.

## Supplementary information

Supplementary information
