# Supplementary material for: Contribution to the ecology of the Italian hare (Lepus corsicanus)
Source: Sci Rep. 2020 Aug 4;10:13071. doi: 10.1038/s41598-020-70013-1 (PMC7403147; doi:10.1038/s41598-020-70013-1)
Supplement: Supplementary file 1 — Supplementary information [file 41598_2020_70013_MOESM1_ESM.pdf]

Supplementary information

Contribution to the ecology of the Italian hare (*Lepus corsicanus*)

Maria Buglione[1], Simona Petrelli[1], Gabriele de Filippo[2], Claudia Troiano[3], Eleonora Rivieccio[1], Tommaso Notomista[1], Valeria Maselli[1], Luciano di Martino[4], Marco Carafa[4], Romano Gregorio[5], Roberta Latini[6], Mario Fortebraccio[7], Giorgia Romeo[8], Claudia Biliotti[9] and Domenico Fulgione[1*]

[1] Department of Biology, University of Naples Federico II, Naples, Italy

[2] Istituto di Gestione della Fauna (IGF), Naples, Italy

[3] Department of Humanities, University of Naples Federico II, Napoli, Italy

[4] Majella National Park, Sulmona, Aquila Italy

[5] Cilento, Vallo di Diano e Alburni National Park, Salerno, Italy

[6] Abruzzo, Lazio and Molise National Park, Pescasseroli, Aquila, Italy

[7] Freelance Forestry Doctor, Potenza, Italy

[8] Wildlife Section, Tuscan Regional Council, Grosseto, Italy

[9]SOS animali Onlus, Wildlife Rescue Center, Semproniano, Grosseto, Italy

Corresponding author:

Domenico Fulgione

Department of Biology, University of Naples Federico II, Via Cinthia 26, 80126 Naples, Italy

E mail address: fulgione@unina.it

Graphical Abstract

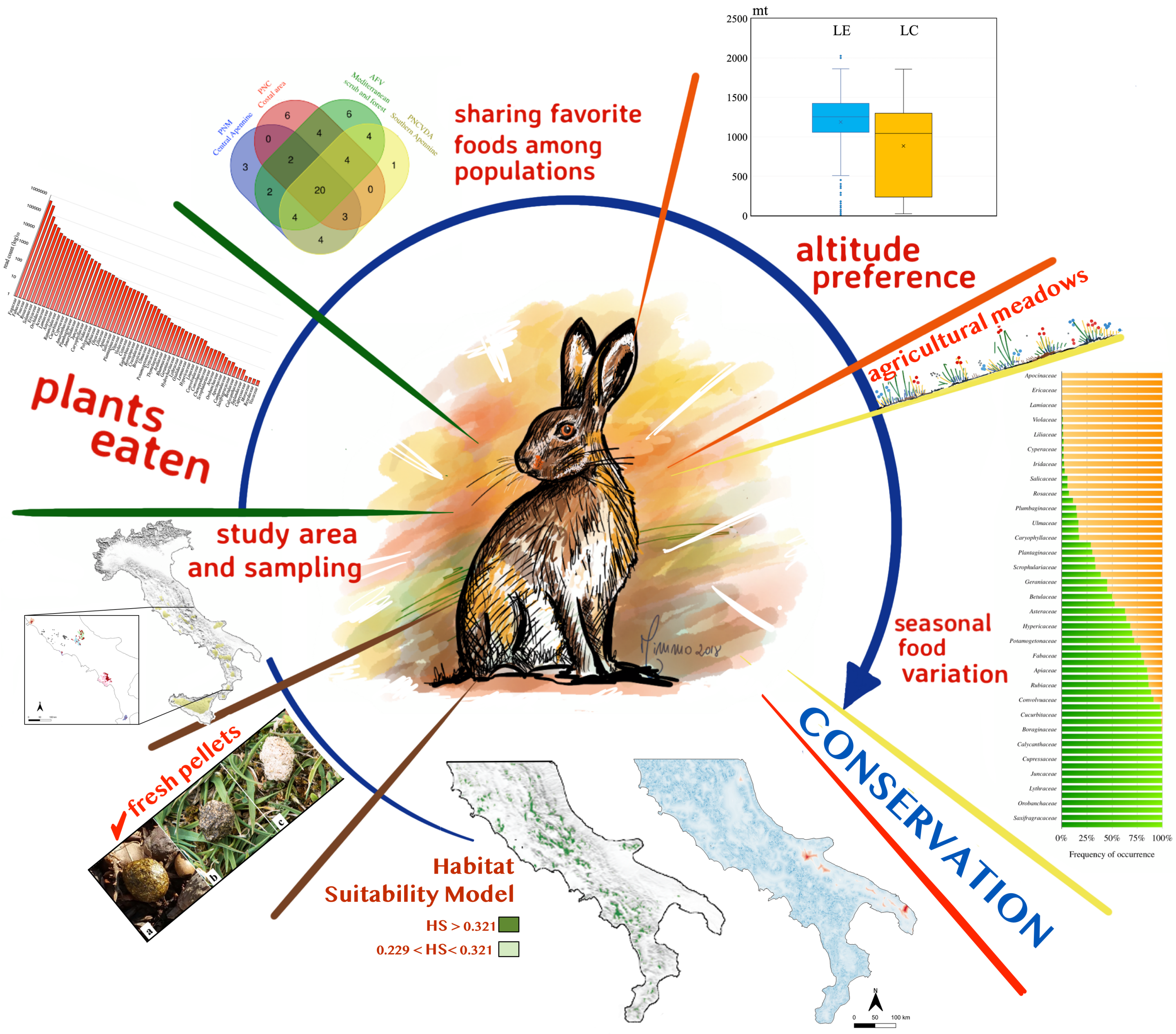

a **b** **c**

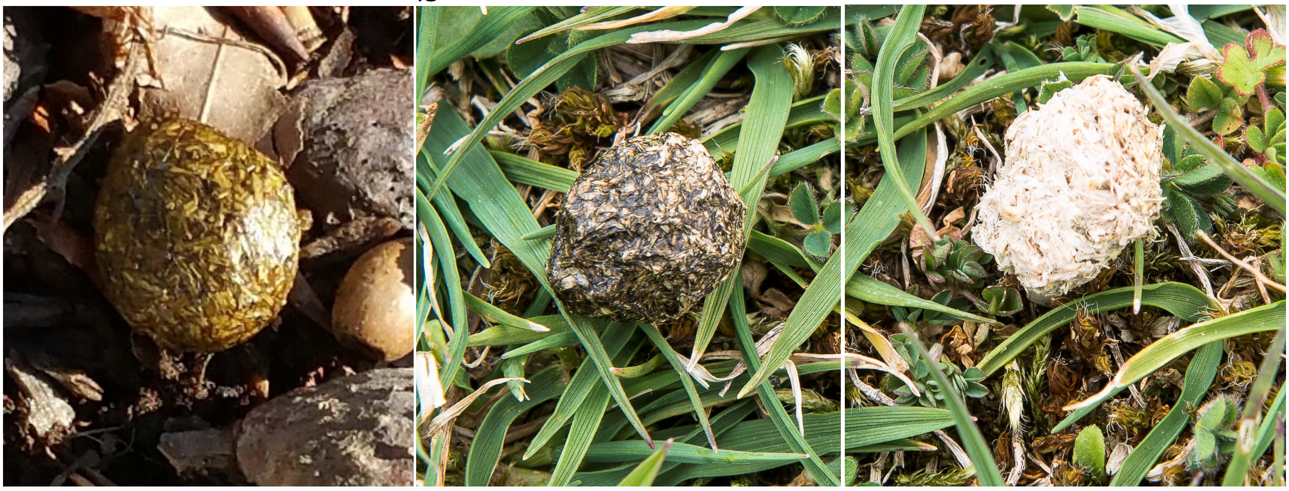

Supplementary Fig. S1. Examples of faecal pellets. Estimate of deposition age by skilled field collectors a) 24 hours; b) 24-48 hours; c) >48 hours.

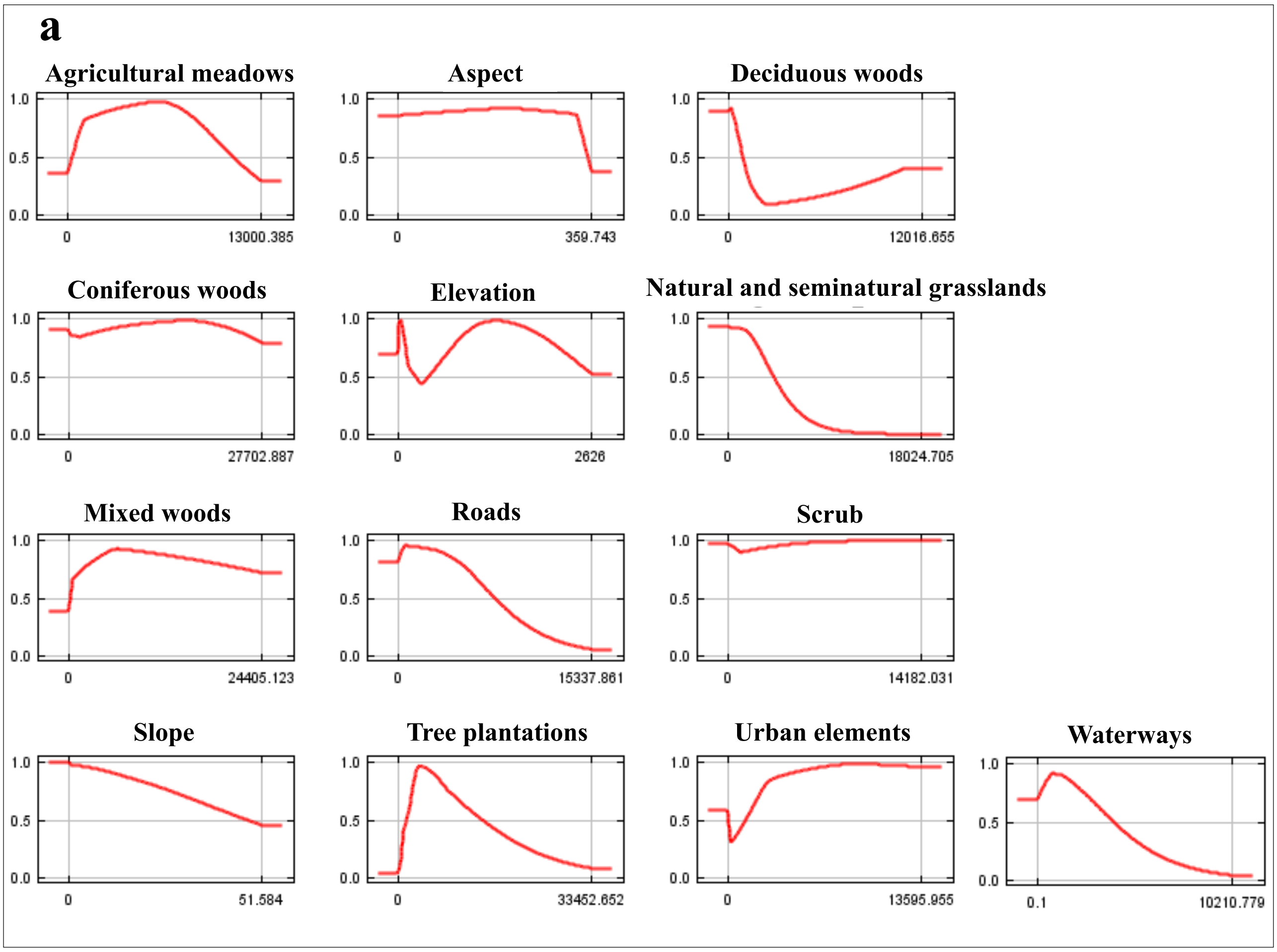

a
Agricultural meadows
Aspect
Deciduous woods
Coniferous woods
Elevation
Natural and seminatural grasslands
Mixed woods
Roads
Scrub
Slope
Tree plantations
Urban elements
Waterways
0
13000.385
359.743
12016.655
27702.887
2626
18024.705
24405.123
15337.861
14182.031
51.584
33452.652
13595.955
0.1
10210.779

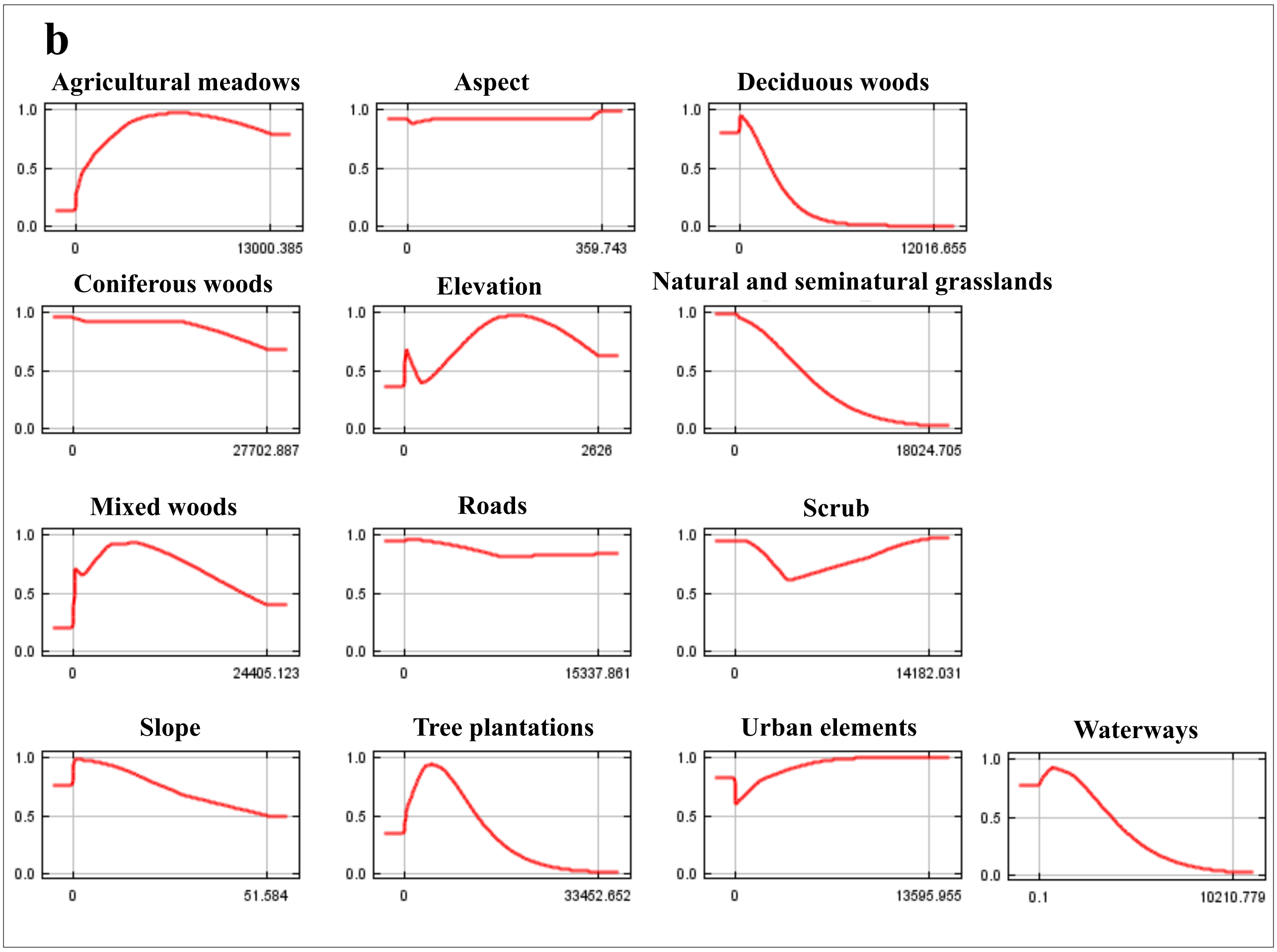

Supplementary Fig. S2. Environmental variable affecting the Maxent prediction. The curves show how the predicted probability of presence changes as each environmental variable is varied, keeping all other environmental variables at their average sample value for a) the Italian hare and b) the European hare.

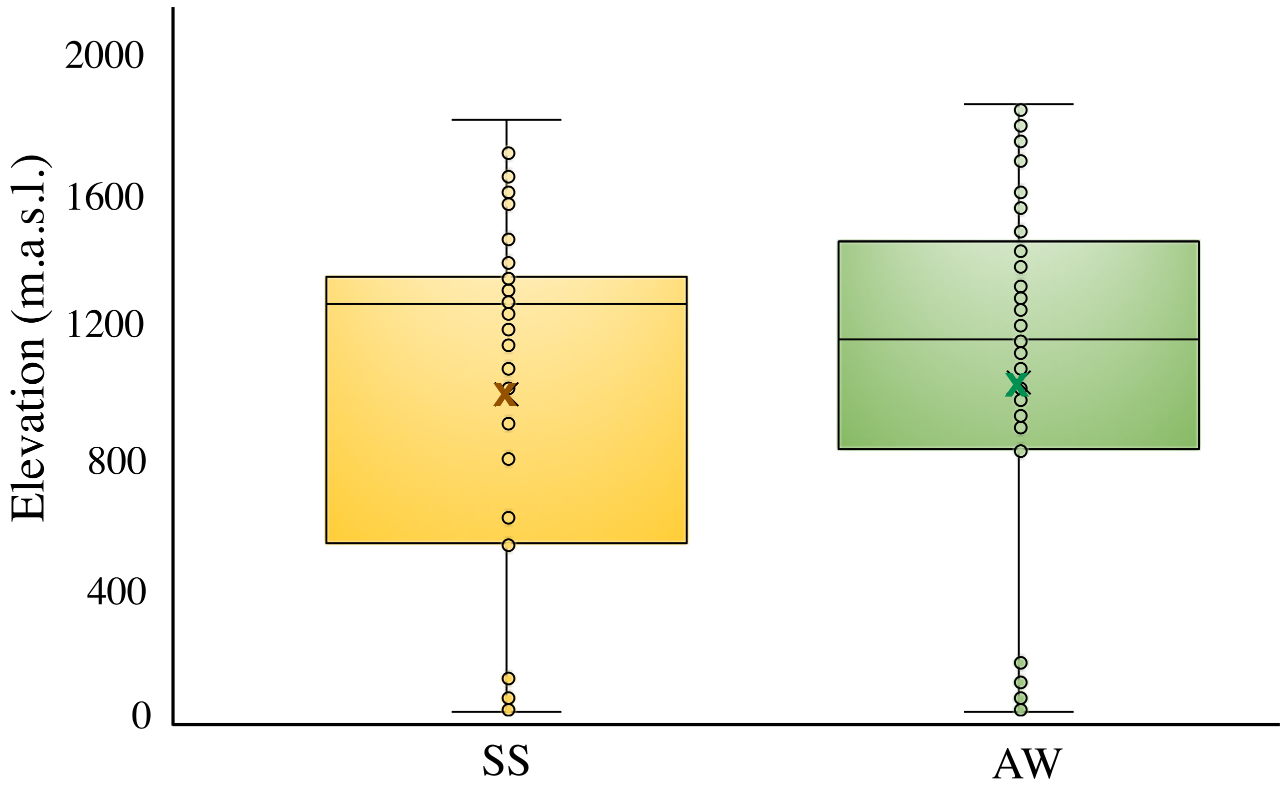

Supplementary Fig. S3. The Italian hare distribution related to the elevation. Observed distribution of the Italian hare in altitudinal range during Spring/Summer (SS) and Autumn/Winter (AW) seasons.

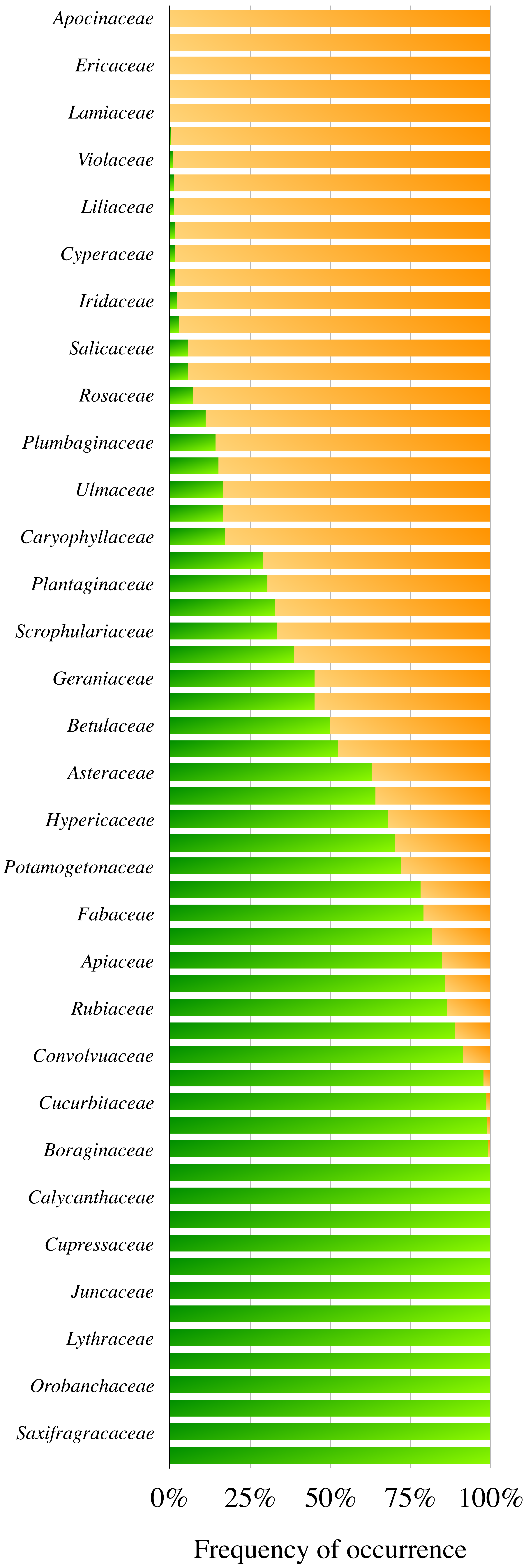

Supplementary Fig. S4. Plant families in seasonal diets of the Italian hare. Frequencies of occurrence of plant families (%) in the diet of the Italian hare during Spring/Summer (green) and Autumn/Winter (yellow) seasons.

Supplementary Table S1. Diet of the Italian hare in Spring/Summer and Autumn/Winter seasons. Qualitative (family/taxon) and quantitative (frequency of occurrence %) data on seasonal diet of the Italian hare according to Spring/Summer and Autumn/Winter seasons.

	Family	Taxon	Frequency of occurrence (%)	
			Spring/Summer	Autumn/Winter
	Fagaceae	*Fagaceae*	3.58502	37.23826
		Fagus_sp.	1.12270	4.10633
		Fagus_sylvatica	0.53671	0.33388
		Quercus_cerris	0.00056	0.00000
		Quercus_ilex	0.00000	0.00019
		Quercus_sp.	0.00149	0.18304
	Fabaceae	*Anthyllis_sp.*	0.30662	0.02651
		Anthyllis_vulneraria	0.00019	0.00000
		Astragalus_sp.	0.00383	0.00000
		Cicer_arietinum	0.00075	0.00065
		Fabaceae	1.92412	0.15513
		Galega_officinalis	0.00056	0.00037
		Hedysarum_coronarium	0.00420	0.00000
		Hedysarum_glomeratum	0.03743	0.20339
		Lathyrus_nissolia	0.00000	0.00019
		Lathyrus_sp.	0.00131	0.00000
		Lotus_sp.	0.02539	0.00513
		Lupinus_angustifolius	0.32510	0.00159
		Medicago_sativa	0.00000	0.00112
		Medicago_sp.	0.01867	0.03818
		Medicago_truncatula	0.01568	0.00252
		Medicago_turbinata	0.00028	0.00000
		Onobrychis_arenaria	0.00000	0.00131
		Onobrychis_caput-galli	0.00037	0.08195
		Onobrychis_montana	0.00019	0.00000
		Onobrychis_sp.	16.53890	5.13456
		Onobrychis_viciifolia	0.00709	0.00131
		Ononis_pusilla	0.00037	0.00000
		Ononis_sp.	0.00028	0.00000
		Ornithopus_compressus	0.00037	0.00000
		Ornithopus_pinnatus	0.00065	0.00000
		Ornithopus_sp.	0.00056	0.00000
		Phaseolus_vulgaris	0.18575	0.00000
		Scorpiurus_vermiculatus	0.00019	0.00000
		Spartium_junceum	0.00037	0.01531
		Trifolium_montanum	0.00019	0.00000
		Trifolium_pallidum	0.13945	0.00000
		Trifolium_pratense	0.00019	0.00000
		Trifolium_repens	0.00784	0.00000
		Trifolium_sp.	3.44324	0.48677
		Trifolium_striatum	0.00019	0.00952
		Trifolium_suffocatum	0.00019	0.00000
		Trifolium_tomentosum	0.00019	0.00000
		Vicia_sp.	0.05302	0.00243
	Poaceae	*Achnatherum_calamagrostis*	0.00056	0.00317
		Agrostis_capillaris	0.00037	0.00187
		Agrostis_sp.	0.00625	0.07570
		Agrostis_stolonifera	0.00000	0.00392
		Alopecurus_myosuroides	0.00280	0.00000
		Alopecurus_sp.	0.00037	0.00000
		Anthoxanthum_alpinum	0.00019	0.00000
		Anthoxanthum_sp.	0.00233	0.00000
		Arrhenatherum_elatius	0.00037	0.00000
		Arrhenatherum_sp.	0.00663	0.00000
		Avena_fatua	0.00019	0.00000
		Avena_sp.	0.10193	0.00112
		Bellardiochloa_variegata	0.00019	0.00000
		Brachypodium_distachyon	0.00280	0.00000
		Brachypodium_retusum	0.00028	0.00784
		Brachypodium_sp.	1.01069	0.01297
		Briza_maxima	0.00000	0.00019
		Bromus_diandrus_var.rigidus	0.00989	0.00149
		Bromus_erectus	0.00131	0.01269
		Bromus_hordeaceus	0.00000	0.00019
		Bromus_sp.	0.59215	0.11481
		Bromus_tectorum	0.00000	0.00075
		Catapodium_sp.	0.00000	0.00037
		Cynodon_sp.	0.00000	0.00056
		Cynosurus_cristatus	0.03771	0.00187
		Cynosurus_echinatus	0.00065	0.00280
		Dactylis_glomerata	0.00037	0.00000
		Dactylis_sp.	0.01148	0.03538
		Dactylorhiza_viridis	0.01083	0.00000

	Family	Taxon	Frequency of occurrence (%)	
			Spring/Summer	Autumn/Winter
		Delairea_odorata	0.00000	0.00019
		Elodea_canadensis	0.00000	0.00019
		Festuca_arundinacea	0.00019	0.00000
		Festuca_circummediterranea	0.00047	0.00392
		Festuca_laevigata	0.02259	0.06515
		Festuca_ovina	0.00000	0.00961
		Festuca_pratensis	0.00019	0.00056
		Festuca_rubra	0.00579	0.00000
		Festuca_sp.	0.13077	0.00579
		Helictochloa_sp.	0.00037	0.00000
		Hordelymus_europaeus	0.00280	0.00000
		Hordeum_sp.	0.00299	0.01540
		Hordeum_vulgare	0.00103	0.00131
		Koeleria_macrantha	0.00149	0.00037
		Koeleria_sp.	0.00971	0.21020
		Lolium_multiflorum	0.01176	0.00000
		Lolium_rigidum	0.00345	0.00000
		Lolium_sp.	0.30634	0.27041
		Luzula_campestris	0.00019	0.00000
		Luzula_sp.	0.00663	0.09931
		Milium_vernale	0.00000	0.00019
		Oryza_sativa	0.00000	0.00075
		Oryza_sativa_Indica_Group	0.00047	0.00000
		Oryza_sativa_Japonica_Group	0.02548	0.00000
		Oryza_sp.	0.00019	0.00196
		Panicum_miliaceum	0.00000	0.00187
		Paspalum_sp.	0.00000	0.00019
		Phalaris_sp.	0.00737	0.00000
		Phleum_alpinum	0.00037	0.00000
		Phleum_pratense	0.07458	0.00019
		Phleum_sp.	0.02464	0.00205
		Poa_bulbosa	0.00000	0.00019
		Poa_compressa	0.00103	0.00000
		Poa_pratensis	0.00000	0.00579
		Poa_sp.	0.52961	0.20283
		Poaceae	3.13223	0.54847
		Psilurus_incurvus	0.00019	0.00000
		Secale_cereale	0.00000	0.00019
		Secale_sp	0.00728	0.00019
		Sesleria_caerulea	0.00121	0.00000
		Setaria_sp.	0.00000	0.00056
		Sporobolus_virginicus	0.00019	0.00000
		Triticum_aestivum	0.00821	0.00560
		Triticum_sp.	0.00177	0.00924
		Zea_mays	0.00336	0.00737
	Rosaceae	*Alchemilla*	0.00271	0.00065
		Cotoneaster_integerrimus	0.00000	0.00019
		Crataegus_sp.	0.00019	0.00243
		Fragaria_vesca_subsp._bracteata	0.08979	0.00000
		Geum_urbanum	0.10883	0.00000
		Malus_domestica	0.00037	0.00000
		Malus_pumila	0.00037	0.00000
		Malus_sp.	0.00047	0.00317
		Potentilla_argentea	0.00056	0.00336
		Potentilla_sp.	0.00625	0.10015
		Prunus_dulcis	0.00000	0.00019
		Prunus_sp.	0.00056	0.17361
		Pyrus_amygdaliformis	0.00000	0.00168
		Pyrus_sp.	0.00103	0.00635
		Rosa_canina	0.00000	0.00411
		Rosa_dumalis	0.00093	0.00000
		Rosa_gallica	0.00075	0.00075
		Rosa_sp.	0.00261	0.04200
		Rosaceae	0.01689	0.00691
		Rubus_sp.	0.00131	2.78014
		Sorbus_sp.	0.00299	0.00000
	Solanaceae	*Capsicum_annuum*	0.00019	0.00000
		Solanaceae	0.00019	0.00000
		Solanum_lycopersicum	0.01381	2.58786
		Solanum_sp.	0.00056	0.00019
		Nicotiana_tabacum	0.00000	0.00019
	Ericaceae	*Arbutus_unedo*	0.00000	1.48122
		Arctostaphylos_sp.	0.00000	0.00037
		Epipactis_atrorubens	0.00112	0.00280
		Erica_carnea	0.00000	0.00019
		Orthilia_secunda	0.00019	0.00000
	Orchidaceae	*Dactylorhiza_incarnata*	0.00019	0.00000
		Dactylorhiza_maculata	0.00019	0.00000

	Family	Taxon	Frequency of occurrence (%)	
			Spring/Summer	Autumn/Winter
		Dactylorhiza_sp.	0.00065	0.00000
		Elymus_caninus	0.07047	0.00047
		Herminium_monorchis	0.00037	0.00000
		Orchidaceae	1.01358	0.00896
		Orchis_italica	0.00019	0.00000
		Orchis_mascula	0.00019	0.00000
		Orchis_provincialis_subsp._pauciflora	0.01857	0.00000
		Orchis_sp.	0.14365	0.00205
	Aceraceae	*Acer_sp.*	0.00056	0.04107
		Acer_campestre	0.00056	0.95786
		Acer_cappadocicum	0.00000	0.00019
		Acer_platanoides	0.00000	0.05078
	Lamiaceae	*Clinopodium_vulgare*	0.00019	0.00000
		Lamarckia_aurea	0.00019	0.00000
		Lamiaceae	0.00037	1.03711
		Prunella_sp.	0.00140	0.00000
		Thymus_pulegioides	0.00056	0.00056
	Asteraceae	*Anthemis_sp.*	0.00019	0.00000
		Artemisia_sp.	0.00056	0.00000
		Asteraceae	0.20871	0.17828
		Bellis_perennis	0.00000	0.00168
		Bellis_sp.	0.00019	0.01269
		Bellis_sylvestris	0.00000	0.00243
		Bidens_frondosa	0.00000	0.09558
		Cichorium_sp.	0.00019	0.00000
		Crepis_sp.	0.28973	0.00261
		Daucus_sp.	0.00000	0.00019
		Galatella_linosyris	0.00000	0.00056
		Gamochaeta_pensylvanica	0.00075	0.00000
		Helminthotheca_echioides	0.00019	0.00000
		Hieracium_racemosum	0.00000	0.00187
		Hypochaeris_achyrophorus	0.00047	0.00000
		Hypochaeris_cretensis	0.00560	0.00093
		Jacobaea_aquatica	0.00000	0.00056
		Picris_sp.	0.00047	0.00019
		Pilosella_sp.	0.00056	0.00000
		Scorzonera_sp.	0.00056	0.00000
		Scorzoneroides_cichoriacea	0.00177	0.00803
		Scorzoneroides_sp.	0.00075	0.00000
		Taraxacum_sp.	0.00187	0.00028
		Tragopogon_sp.	0.00401	0.00000
	Ranunclulaceae	*Adonis_vernalis*	0.00187	0.00000
		Clematis_vitalba	0.00327	0.76296
		Ficaria_verna_subsp.bulbilifera	0.00019	0.00000
		Ranunculaceae	0.00149	0.00000
		Ranunculus_sp.	0.00261	0.00019
	Cucurbitaceae	*Bryonia_dioica*	0.12508	0.00019
		Cucumis_melo	0.44841	0.00261
		Cucumis_sp.	0.00131	0.00523
		Cucurbita_pepo	0.00205	0.00000
		Cucurbitaceae	0.00093	0.00000
		Lagenaria_siceraria	0.02632	0.00000
	Cyperaceae	*Bolboschoenus_maritimus*	0.00000	0.00103
		Carex_brachystachys	0.00056	0.00000
		Carex_echinata	0.00000	0.00019
		Carex_sp.	0.00747	0.13656
		Cyperaceae	0.00000	0.40276
		Isolepis_cernua	0.00093	0.00000
	Amaranthaceae	*Alternanthera_philoxeroides*	0.00019	0.00000
		Amaranthus_hybridus	0.00000	0.00028
		Amaranthus_spinosus	0.00000	0.00019
		Beta_sp.	0.00000	0.13534
		Beta_vulgaris	0.00691	0.00037
		Digitalis_sp.	0.00093	0.00000
	Plumbaginaceae	*Armeria_canescens*	0.00952	0.05600
		Armeria_sp.	0.02866	0.17959
		Limonium_narbonense	0.00037	0.00000
	Amaryllidaceae	*Allium_cepa*	0.00084	0.00000
		Allium_sp.	0.23484	0.00541
		Allium_vineale	0.00019	0.00000
	Juncaceae	*Juncus_bufonius*	0.00672	0.00000
		Juncus_capitatus	0.00131	0.00000
		Juncus_effusus	0.00075	0.00000
		Juncus_ranarius	0.00019	0.00000
		Juncus_sp.	0.20712	0.00000
	Caryophyllaceae	*Caryophyllaceae*	0.01437	0.09343
		Chaerophyllum	0.00112	0.00000
		Deschampsia_cespitosa	0.00056	0.00000

	Family	Taxon	Frequency of occurrence (%)	
			Spring/Summer	Autumn/Winter
		Silene_italica	0.00028	0.00131
		Silene_sp.	0.00429	0.00019
		Stellaria_media	0.00000	0.00037
		Stellaria_sp.	0.00000	0.00037
	Iridaceae	*Crocus_imperati*	0.00019	0.00000
		Crocus_sp.	0.00056	0.00000
		Iridaceae	0.00019	0.11238
		Romulea_bulbocodium	0.00187	0.00168
	Polygonaceae	*Bistorta_sp.*	0.00635	0.00000
		Fagopyrum_esculentum	0.00019	0.00000
		Polygonaceae	0.00037	0.00000
		Polygonum_aviculare	0.00037	0.00000
		Polygonum_sp.	0.07169	0.01176
		Rumex_acetosa	0.00373	0.00728
		Rumex_sp.	0.00579	0.00093
	Rubiaceae	*Asperula_sp.*	0.06552	0.00168
		Rubia	0.00243	0.00019
		Rubiaceae	0.01708	0.01176
	Oleaceae	*Fraxinus_sp.*	0.00093	0.00019
		Oleaceae	0.00000	0.00476
		Phillyrea_latifolia	0.00000	0.00037
		Phillyrea_sp.	0.00037	0.07449
	Liliaceae	*Asparagus_acutifolius*	0.00000	0.00019
		Asparagus_officinalis	0.00000	0.00019
		Asparagus_sp.	0.00056	0.07299
		Gagea_bohemica	0.00037	0.00000
		Gagea_lutea	0.00000	0.00037
	Apiaceae	*Apiaceae*	0.06011	0.00373
		Bupleurum__sp.	0.00000	0.00112
		Capsella_sp.	0.00037	0.00000
		Dasypyrum_villosum	0.00495	0.00373
		Oenanthe_sp.	0.00065	0.00000
		Peucedanum_officinale	0.00075	0.00597
	Salicaceae	*Populus_sp.*	0.00243	0.03920
		Populus_tremula_x_Populus_alba	0.00037	0.00448
		Salix_sp.	0.00000	0.00336
	Plantaginaceae	*Dianthus_sp.*	0.00000	0.00149
		Plantaginaceae	0.00056	0.00000
		Plantago_maritima	0.00056	0.00000
		Plantago_sp.	0.01148	0.03090
	Vitaceae	*Vitis_vinifera*	0.01409	0.02875
	Violaceae	*Viola_sp.*	0.00037	0.01120
		Viola_tricolor	0.00000	0.02968
	Cistaceae	*Cistaceae*	0.00112	0.03808
	Euphorbiaceae	*Ericaceae*	0.00131	0.05638
		Erophila_sp.	0.00019	0.00000
		Euphorbia_esula	0.00000	0.00187
		Euphorbia_helioscopia	0.00513	0.00000
		Euphorbia_marginata	0.00411	0.00000
		Euphorbia_nicaeensis	0.00000	0.00019
		Euphorbia_sp.	0.02137	0.00317
		Ricinus_communis	0.00019	0.00000
	Boraginaceae	*Boraginaceae*	0.01708	0.00000
		Dysphania_botrys	0.00000	0.25762
		Myosotis_sp.	0.00047	0.00000
		Pulmonaria_obscura	0.00243	0.00000
	Crassulaceae	*Crassulaceae*	0.00131	0.00093
		Sedum_rupestre	0.00000	0.00019
		Sedum_sp.	0.00467	0.00840
	Brassicaceae	*Aethionema_saxatile*	0.00000	0.00019
		Arabidopsis_thaliana	0.00467	0.00252
		Brassica_rapa	0.00019	0.00000
		Brassica_sp.	0.00131	0.00196
		Brassicaceae	0.00075	0.00327
		Sisymbrium_orientale	0.00000	0.00047
	Ulmaceae	*Ulmus_sp.*	0.00205	0.01036
	Potamogetonaceae	*Potamogeton_nodosus*	0.00000	0.00224
		Potamogeton_sp.	0.00019	0.00000
		Stuckenia_pectinata	0.00560	0.00000
	Urticaceae	*Urtica_sp.*	0.00411	0.00373
	Thymelaeaceae	*Thymelaea_passerina*	0.00037	0.00000
		Thymelaeaceae	0.00000	0.00616
	Araliaceae	*Hedera_helix*	0.00037	0.00000
		Hedera_sp.	0.00084	0.00299
	Rhamnaceae	*Ziziphus_jujuba*	0.00056	0.00317
	Geraniaceae	*Geraniaceae*	0.00019	0.00000
		Geranium_sp.	0.00131	0.00205
		Geranium_tuberosum	0.00019	0.00000

	Family	Taxon	Frequency of occurrence (%)	
			Spring/Summer	Autumn/Winter
	Convoluaceae	*Convoluaceae*	0.00019	0.00103
	Convolvulaceae	*Calystegia_sepium*	0.00196	0.00019
	Hydrocharitaceae	*Echium_candicans*	0.00000	0.00019
		Najas_marina	0.00299	0.00019
	Lythraceae	*Lythraceae*	0.00289	0.00000
	Oxalidaceae	*Oxalis_sp.*	0.00196	0.00084
	Lemnaceae	*Wolffia_arrhiza*	0.00280	0.00000
	Hypericaceae	*Hypericum_montanum*	0.00093	0.00000
		Hypericum_sp.	0.00084	0.00084
	Linaceae	*Linum_sp.*	0.00000	0.00243
	Chenopodiaceae	*Chenopodi_sp.*	0.00000	0.00075
		Salicornia_patula	0.00019	0.00019
	Scrophulariaceae	*Veronica_cymbalaria*	0.00019	0.00000
		Veronica_sp.	0.00019	0.00075
	Araceae	*Araceae*	0.00112	0.00000
	Orobanchaceae	*Orobanche_sp.*	0.00103	0.00000
	Apocinaceae	*Nerium_oleander*	0.00000	0.00075
	Campanulaceae	*Campanula_sp.*	0.00056	0.00000
	Saxifragracaceae	*Saxifraga*	0.00056	0.00000
	Betulaceae	*Alnus_sp.*	0.00019	0.00000
		Betula_pendula	0.00000	0.00019
	Calycanthaceae	*Calycanthus_floridus*	0.00037	0.00000
	Juglandaceae	*Juglans_regia*	0.00037	0.00000
	Cupressaceae	*Cupressaceae*	0.00019	0.00000
	Moraceae	*Ficus_carica*	0.00019	0.00000
	Resedaceae	*Reseda*	0.00019	0.00000
	Viscaceae	*Viscum_album*	0.00019	0.00000
**Total**	**62**	**334**	**37.95558**	**62.04442**

Supplementary Table S2. Details of Venn Diagram for diet of the Italian hare in different environmental typologies. Details of shared and unique plant families in the diets of the Italian hare samples collected in Cilento, Vallo di Diano e Alburni National Park (PNCVDA), Majella National Park (PNM), Circeo National Park (PNC) and Game reserve (AFV).

**Names**	**Total**	**Plant family**
**AVF-PNC-PNCVDA-PNM**	20	*Geraniaceae Aceraceae Vitaceae Solanaceae Caryophyllaceae Apiaceae Fagaceae Urticaceae Cucurbitaceae Poaceae Orchidaceae Rosaceae Fabaceae Polygonaceae Rubiaceae Asteraceae Liliaceae Salicaceae Cyperaceae Euphorbiaceae*
**AVF-PNC-PNM**	2	*Chenopodiaceae Plantaginaceae*
**PNC-PNCVDA-PNM**	3	*Lamiaceae Boraginaceae Violaceae*
**AVF-PNCVDA-PNM**	4	*Brassicaceae Plumbaginaceae Araliaceae Potamogetonaceae*
**AVF-PNC-PNCVDA**	4	*Amaranthaceae Ericaceae Scrophulariaceae Oleaceae*
**AVF-PNM**	2	*Amaryllidaceae Thymelaeaceae*
**PNCVDA-PNM**	4	*Crassulaceae Ranunclulaceae Rhamnaceae Iridaceae*
**AVF-PNC**	4	*Juncaceae Hypericaceae Oxalidaceae Ulmaceae*
**AVF-PNCVDA**	4	*Linaceae Cistaceae Convoluaceae Betulaceae*
**PNM**	3	*Saxifragracaceae Resedaceae Viscaceae*
**PNC**	6	*Convolvulaceae Lythraceae Juglandaceae Orobanchaceae Hydrocharitaceae Araceae*
**AVF**	6	*Cupressaceae Lemnaceae Campanulaceae Ranunculaceae Moraceae Calycanthaceae*
**PNCVDA**	1	*Apocinaceae*

Supplementary Table S3. Details of Venn Diagram for seasonal diet of the Italian hare. Details of shared (SS-AW) and unique (SS and AW) plant families in Spring/Summer (SS) and Autumn/Winter (AW) diets.

SS-AW	SS	AW
Aceraceae	*Araceae*	*Apocinaceae*
Amaranthaceae	*Calycanthaceae*	*Linaceae*
Amaryllidaceae	*Campanulaceae*	
Apiaceae	*Cupressaceae*	
Araliaceae	*Juglandaceae*	
Asteraceae	*Juncaceae*	
Betulaceae	*Lemnaceae*	
Boraginaceae	*Lythraceae*	
Brassicaceae	*Moraceae*	
Caryophyllaceae	*Orobanchaceae*	
Chenopodiaceae	*Resedaceae*	
Cistaceae	*Saxifragracaceae*	
Convoluaceae	*Viscaceae*	
Convolvulaceae		
Crassulaceae		
Cucurbitaceae		
Cyperaceae		
Ericaceae		
Euphorbiaceae		
Fabaceae		
Fagaceae		
Geraniaceae		
Hydrocharitaceae		
Hypericaceae		
Iridaceae		
Lamiaceae		
Liliaceae		
Oleaceae		
Orchidaceae		
Oxalidaceae		
Plantaginaceae		
Plumbaginaceae		
Poaceae		
Polygonaceae		
Potamogetonaceae		
Ranunclulaceae		

	Rhamnaceae			
	Rosaceae			
	Rubiaceae			
	Salicaceae			
	Scrophulariaceae			
	Solanaceae			
	Thymelaeaceae			
	Ulmaceae			
	Urticaceae			
	Violaceae			
	Vitaceae			
**Total**		47	13	2
